# Influences of Salt Stress on Cotton Metabolism and Its Consequential Effects on the Development and Fecundity of *Aphis gossypii* Glover

**DOI:** 10.3390/insects15090713

**Published:** 2024-09-18

**Authors:** Wangquan Jiao, Bingmei Song, Hongsheng Pan, Xiaoning Liu

**Affiliations:** 1 Xinjiang Key Laboratory of Biological Resources and Genetic Engineering, College of Life Science and Technology, Xinjiang University, Urumqi 830046, China; 18770031425@163.com (W.J.); songbingmei1999@163.com (B.S.); 2 National Plant Protection Scientific Observation and Experiment Station of Korla, Institute of Plant Protection, Xinjiang Academy of Agricultural Sciences, Urumqi 830091, China

**Keywords:** cotton aphids, growth and development, NaCl stress, metabolome, differential metabolites

## Abstract

**Simple Summary:**

In this study, 0 mM NaCl was used as the control, while 75 mM NaCl (4.4‰ salt content) and 150 mM NaCl (8.8‰ salt content) were employed to simulate mild and moderate salinized soil environments, respectively. The differences in the metabolome of cotton plants under different salt stress conditions and the fitness of cotton aphids (*Aphis gossypii* Glover) were analyzed. The results demonstrated a significant decrease in fecundity, adult longevity, and survival rate of cotton aphids on salt-stressed cotton plants with increasing NaCl concentration. Furthermore, it was observed that higher concentrations of NaCl led to the upregulation of 49 metabolites and the downregulation of 86 metabolites closely associated with the growth, development, and fecundity of cotton aphids. Therefore, these substances present in cotton leaves play a crucial role as mediators influencing the growth and development of cotton aphids.

**Abstract:**

The degree of global soil salinization is gradually deepening, which will inevitably affect agricultural ecology. It has been found that salt stress induces the resistance of host plants to phytophagous pests. However, little is known about the effects of salt-stressed cotton plants on the fitness of cotton aphids (*Aphis gossypii* Glover). In this study, we investigated the differences between cotton metabolomes under mild (75 mM NaCl) and moderate (150 mM NaCl) salinity conditions and their effects on the fitness of cotton aphids. The results showed that 49 metabolites exhibited significant upregulation, while 86 metabolites were downregulated, with the increasing NaCl concentration. The duration of nymphal aphids under 150 mM NaCl significantly extended to 6.31 days when compared with the control (0 mM NaCl, 4.10 days). Meanwhile, the longevity of adult aphids decreased significantly under 75 and 150 mM NaCl, with an average of 10.38 days (0 mM NaCl) reduced to 8.55 and 4.89 days, respectively. Additionally, the total reproduction number of single females decreased from 31.31 (0 mM NaCl) to 21.13 (75 mM NaCl) and 10.75 (150 mM NaCl), whereas the survival rate of aphids decreased from 81.25% (0 mM NaCl) to 56.25% (75 mM NaCl) and 34.38% (150 mM NaCl) on the 12th day. These results support the hypothesis that plants growing under salt stress are better defended against herbivores. Furthermore, 49 differential metabolites were found to be negatively correlated with the longevity and fecundity of adult aphids, while 86 different metabolites showed the opposite trend. These results provide insights into the occurrence and control of cotton aphids amidst the escalating issue of secondary salinization.

## 1. Introduction

Cotton (*Gossypium* spp.), the second largest crop after food, is the most important fiber and oil crop, accounting for 35% of the total global fiber consumption [1]. Cotton yield is limited by abiotic stresses (including cold, high temperature, drought, salt, or chemical pollutants) and biotic stresses (including cotton bollworm, cotton aphid, cotton mite, etc.). Many studies have shown that environmental abiotic factors lead to changes in plant nutrients and defensive chemicals, which have a bottom–up effect on herbivorous pests [2].

Soil salinization is a global agricultural problem, which has adverse effects on plant growth and crop yield. Based on the soluble salt content (g/kg), salty soil can be categorized into the following five classes: non-salinized (<3‰), slightly saline (3~6‰), moderately saline (6~10‰), severely saline (10~20‰), and saline soil (>20‰) [3]. According to the FAO, salt-affected soils globally encompass an area of 833 million hectares, accounting for approximately 7% of the total land and 33% of cropland worldwide [4,5], and it is estimated to cause direct losses to global agriculture of about $27.3 billion per year [6]. Furthermore, due to the excessive utilization of chemical fertilizers, improper irrigation practices, industrial pollution, gradual depletion of marine resources, and mineral weathering processes, salinized land is expanding at a rate of 10% annually [5]. It is estimated that nearly 50% of global cropland will be affected by salinization by 2050 [7,8].

In addition, biotic stress is one of the main factors endangering global crop production. It is estimated that the field losses caused by pests have exceeded 10%, and a figure that rises to 50–80% in the absence of control measures [9]. Cotton aphids, *Aphis gossypii* Glover (Insecta, Homoptera, Aphididae) have strong fecundity and mainly damage more than one hundred crops such as cotton, melons, soybeans, jute, potato, and sweet potato [10]. With the widespread cultivation of transgenic *Bt* cotton, cotton bollworms have been effectively controlled while aphids have emerged as the major pest [11]. Cotton aphids, the main piercing–sucking pest on cotton, aggregate as adults and nymphs on the undersides of cotton leaves and twigs to feed on sap, resulting in leaf curling, stunted plant growth, reduced height, fewer fruit branches, and decreased foliage [12]. Meanwhile, the honeydew secreted by cotton aphids can promote mold proliferation and seriously affect both the yield and quality of cotton [13]. Statistics show that Xinjiang’s cotton fields had been affected by pests and diseases over an area of 86,333 hectares by the end of July 2019, leading to a decrease in cotton yield ranging from 15% to 20%, primarily due to infestations by cotton aphids [14].

However, plants have evolved complex defense networks in response to biotic and abiotic stresses, and studies have also reported that abiotic factors can have a bottom–up effect on herbivorous pests by affecting host plants [15]. Excessive soil salinity induces osmotic stress and ion toxicity in plants, leading to reduced growth rate, diminished plant height, decreased branching patterns, and leaf wilting [16]. Moreover, it also increases soil water potential impeding water and nutrient absorption by plants, resulting in reduced leaf turgor pressure. Consequently, these effects ultimately lead to stomatal closure, negatively impacting photosynthesis [5,17]. High levels of sodium (Na^+^) in the soil environment affect calcium (Ca^2+^) and potassium (K^+^) uptake by plants, leading to an imbalance in the K^+^/Na^+^ ratio, thereby disrupting ion homeostasis within the plant system [18]. Soil salt stress diminishes cotton nitrogen uptake and transport and significantly reduces plant growth, water consumption, leaf water, and leaf gas exchange rates [19]. The contents of amino acids, sugars, and abscisic acid increased, and the contents of vitamins and terpenoids decreased. Genes in metabolic pathways such as flavonoid and amino acid biosynthesis were continuously upregulated [20]. It has been reported that when the yield of cotton cultivated areas in the Akcakale district in 2009 was calculated according to the effect of salinity, 1,840,625 kg of yield losses had occurred due to salinity, and the resulting income loss was USD 935,711 [21].

The defenses evolved by host plants in response to herbivorous pests include chemicals (such as phenolic compounds, alkaloids, terpenoids, volatile organic compounds, and extrafloral nectar), the ability to directly produce toxic effects on herbivorous pests or attract natural enemies, and the fact that plant structures (such as trichomes, spines) can reduce the damage of pests [22]. Interestingly, the increased primary and secondary metabolism of plants in response to salt stress is also an important chemical substance for defense against herbivorous pests. Therefore, it is also very interesting to explore the interactions among salt stress, plants, and pests. The content of phenolic acids increased by 34.63% and 30.91%, while the content of tannin increased by 40.63% and 34.87%, in the SCRC28 and K836 cotton treated with 200 mM NaCl. Furthermore, the development duration of the immature stages under the 200 mM NaCl treatments of SCRC28 and K836 increased by 12.84% and 11.94%, relative to the control, respectively. The fecundity of single females feeding on salt-stressed (200 mM NaCl) SCRC28 and K836 reduced by 31.96% and 36.16%, respectively [23]. In addition, this phenomenon was also found under drought stress, i.e., the water content, aboveground biomass, and nitrogen content of cotton plants decreased, the content of soluble protein, soluble sugar, and tannin increased, and the fecundity and population richness of *A. gossypii* decreased significantly [24]. Furthermore, it has been observed through a literature analysis that salt-stressed cotton plants can effectively suppress aphid infestation; higher salinity levels corresponded to lower occurrence rates [25]. Nevertheless, limited knowledge exists regarding the impact of salt stress on the growth, development, and fecundity of cotton aphids by influencing host plants.

This study aimed to analyze the effects of different degrees of soil salinization on cotton plant metabolites, as well as the growth, development, and fecundity of cotton aphids. However, our previous studies found that cotton plants have a large number of deaths and antifeedant effects on cotton aphids under the condition of 225 mM NaCl (salt content 13.2‰), simulating severe salinized soil. Therefore, only two treatments of mild (75 mM NaCl, salt content 4.4‰) and moderate (150 mM NaCl, salt content 8.8‰) were retained. Furthermore, the relationship between different metabolites in cotton plants and the fitness of cotton aphids under salt stress conditions was examined. Ultimately, this research elucidated the effects of salt stress on cotton metabolism and its consequences on the development and fecundity of cotton aphids. These findings will provide an important theoretical basis for the occurrence and control of cotton aphids amidst the escalating problem of secondary salinization.

## 2. Materials and Methods

### 2.1. Plants Culture and Salt Stress

Cotton (*Gossypium hirsutum* L. var. CCRI49) seeds were sown in plastic pots (top diameter: 120 mm, bottom diameter: 90 mm, height: 100 mm) filled with a mixture of peat, vermiculite, and field soil (volume ratio: 6:1:1) in a greenhouse at 26 ± 1 °C, 50 ± 5% RH and 16 h:8 h (L:D) photoperiod. The plants were cultivated in these pots until reaching the two-true-leaf stage. Subsequently, plants exhibiting consistent growth were carefully selected and transplanted into black plastic boxes (diameter: 15 cm, height: 20 cm), each containing 1 L of Hoagland nutrient solution [24]. The nutrient solution was replenished with fresh medium weekly. Each individual plant was accommodated in a separate black plastic box, which was equipped with an oxygen pump for daily ventilation lasting 3–4 h. At the five-leaf stage, seedings demonstrating robust growth were chosen for subjecting to salt stress treatments. In our experimental design, three NaCl concentrations (0 as control, 75 [4.4‰] mM NaCl, and 150 [8.8‰] mM NaCl) were employed to simulate varying degrees of salt stress on cotton plants, where 75 and 150 mM represent mild and moderate levels of salinity stress, respectively. All treated plants within the experiment were collectively maintained in a greenhouse at 26 ± 1 °C, 50% ± 5%RH and 16 h:8 h (L:D) photoperiod.

### 2.2. Effects of Salt Stress on Cotton Metabolomics

#### 2.2.1. Sampling

After subjecting cotton plants to NaCl stress treatment for a duration of two weeks during the seeding stage, 30 cotton plants (3 biological replicates, 10 cotton plants per replicate) were randomly selected from each treatment group. The second leaf from the top was carefully collected and placed into 10 mL sampling tubes as experimental materials. Subsequently, the samples were immediately frozen in liquid nitrogen and stored at −80 °C.

#### 2.2.2. Analysis of Cotton Metabolomics

After vacuum freeze-drying (CentriVap, Labconco, Kansas City, MO, USA) three replicate cotton leaf samples, 50 mg was weighed (QUINTIX244-1CN, Sartorius, Gottingen, Germany) from each sample and 1000 μL of extraction solution (methanol–acetonitrile–water in a 2:2:1 ratio) was added; the mixture was then vortexed (TL2020, Bionoon, Beijing, China) for 30 s. After that, the samples were processed with a grinder (TL-3000, Bionoon, Beijing, China) at 45 Hz for 10 min and sonicated (XM-P22H, Xiaomei Ultrasonic Instruments Co., Ltd., Kunshan, China) for 10 min in an ice water bath. The samples were then left to stand at −20 °C for 1 h, followed by centrifugation at 4 °C, 7100× *g* (GL0650R, Monad, Suzhou, China) for 15 min, and the supernatant was dried in a vacuum concentrator (CV200, Jiaimu Technology Co., Ltd., Beijing, China). To improve the quality of the cotton metabolites, 160 μL of extraction solution (acetonitrile–water volume ratio = 1:1) was added to the dried metabolites for resuspension, followed by another round of vortexing for 30 s and sonication for 10 min in an ice water bath. Subsequently, the samples were centrifuged at 4 °C, 7100× *g* for 15 min, and the supernatant was collected. Finally, take 10 μL of supernatant from each replicate sample and mix to form a QC sample for LC–MS/MS analysis.

The sample extracts were analyzed using a UPLC-ESI-MS/MS system, UPLC (Waters Acquity I-Class PLUS, Waters Corporation, 34 Maple St., Milford, MA 01757 USA); MS (Applied Biosystems QTRAP 6500+, AB SCIEX, Marsiling Industrial Estate, Singapore). The analytical conditions were as follows: UPLC column, water, and HSS-T3 (1.8 µm, 2.1 mm × 100 mm); the mobile phase consisted of solvent A, which was pure water with 0.1% formic acid and 5 mM ammonium acetate; and solvent B, which was acetonitrile with 0.1% formic acid. Sample measurements were performed using a gradient program that initiated at the starting conditions of 98% A and 2% B for a duration of 1.5 min. Within a span of 5.0 min, a linear gradient to reach a composition of 50% A and 50% B was programmed. Subsequently within another span of time lasting for 9 min, a linear gradient to achieve the composition of 2% A and 98% B was programmed, and this composition was kept for 1 min. Finally, a composition of 98% A and 2% B was set within 1 min and kept for 3 min. The flow velocity was set as 0.35 mL per minute; the column oven was set to 50 °C; the injection volume was 2 μL. The effluent was alternatively connected to an ESI-triple quadrupole–linear ion trap (QTRAP)–MS.

The operation parameters of the ESI source were as follows: the source temperature was set at 550 °C; the ion spray voltage (IS) was adjusted to 5500 V in positive ion mode and −4500 V in negative ion mode; the gas pressures for ion source gas I (GSI), gas II (GSII), curtain gas (CUR) were set at 50, 55, and 35 psi, respectively; medium collision-activated dissociation (CAD) was employed. Instrument tuning and mass calibration were performed using polypropylene glycol solutions with concentrations of 0.01 mM and 0.1 mM in the triple quadrupole (QQQ) and linear ion trap (LIT) modes, respectively. QQQ scans were acquired as MRM experiments with nitrogen collision gas set to medium pressure. Declustering potential (DP) and collision energy (CE) for individual MRM transitions underwent further optimization through DP and CE adjustments. A specific set of MRM transitions were monitored during each period according to the eluted metabolites within that period [26].

### 2.3. Aphis gossypii

Cotton aphids come from 1 parthenogenetic female aphid collected from cotton field at the National Plant Protection Scientific Observation and Experiment Station in Korla (Korla, Bayingol Mongolian Autonomous Prefecture, Xinjiang Uygur Autonomous Region, China; 41.75° N, 85.81° E). The aphids were reared on 4 to 5 leaf stage potted cotton plants in a climate-controlled growth chamber with 26 ± 1 °C, 50 ± 5% RH, and 16:8 (L/D) h photoperiod. Cotton plants in sampled fields had been maintained without any pesticide applications before aphid collection.

### 2.4. Effects of Salt-Stressed Cotton Plants on Growth, Development and Fecundity of A. gossypii

To investigate the effects of salt stress conditions on cotton aphids at the individual level, two apterous adult aphids were placed on the second true leaf from the top of cotton plants treated with NaCl at one of three concentrations for a duration of six days. Additionally, the leaves were covered with 0.125 mm diameter mesh bags to prevent aphids from escaping. After 12 h, the adult aphids were removed, leaving only one first instar nymph per leaf to measure their response to salt-stressed cotton plants. In this experiment, 30 replicates were designed for each treatment group. Each cotton plant was infested by only one nymph, and survival rate as well as developmental times were recorded every 12 h. Offspring produced by molting adults were recorded daily and then removed until all adults died.

### 2.5. Statistical Analysis

Microsoft Excel 2021 (Microsoft Corporation, Redmond, WA, USA), SPSS 20.0 (IBM, New York, NY, USA), and GraphPad prism 8.3.0 (GraphPad Software, San Diego, CA, USA) were used for data statistics, analysis, and graphic creation. Adult longevity, total longevity, nymph duration (transformed by cosine function), and adult fecundity (transformed by square root function) all exhibited normal distribution and homogeneity of variance as determined by the one-way ANOVA test. Multiple comparisons were conducted using Tukey’s method. However, the nymph duration data from first to fourth instar did not follow a normally distribution, thereby the Kruskal–Wallis H (K) test was employed followed by pairwise comparisons using the Mann–Whitney U test. Furthermore, survival rate data were analyzed using the Kaplan–Meier method with significant differences among the treatment groups assessed through log-rank testing.

Based on the grouping information, the multiple difference were calculated and compared, and a T test was used to calculate the significance (*p* value) of each compound. The OPLS-DA modeling was performed using the R language package ropls, with 200 permutation tests conducted to validate the model’s reliability. The VIP value of the model was calculated using multiple cross-validation. By integrating the difference multiples, *p* value and VIP value from the OPLS-DA model, differential metabolites were screened based on the criteria including *FC* > 1, *p* value < 0.05, and VIP > 1. Additionally, a hypergeometric distribution test was used to evaluate KEGG pathway enrichment significance for these differentially expressed metabolites.

## 3. Results

### 3.1. Quality Control and PCA Analysis of Total Samples

**Figure 1:**
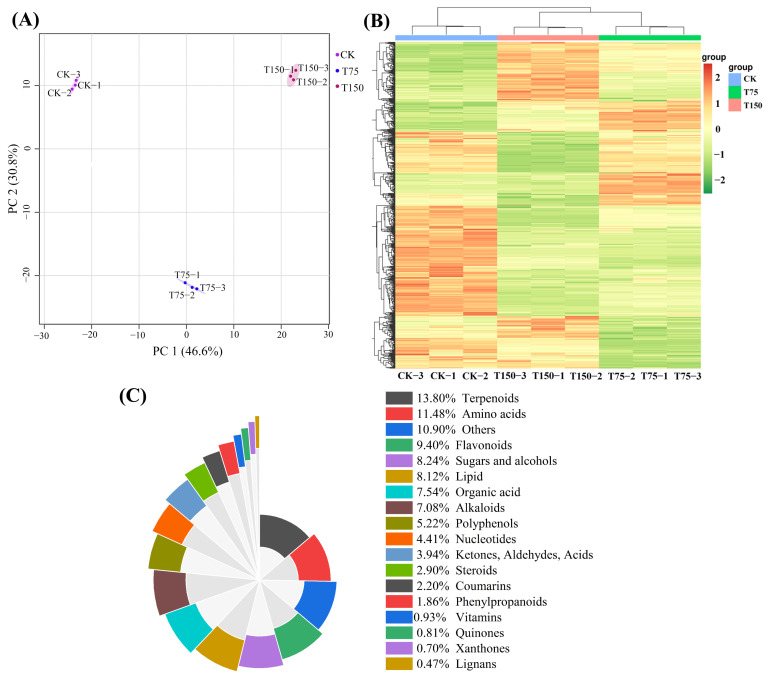
All metabolites of cotton plants under salt stress. (**A**) Principal component analysis results for metabolomics data. (**B**) The heatmap showed the results of the clustering analysis for metabolites. (**C**) The classification of metabolites in response to salt stress.

The data of cotton metabolome were analyzed using principal component analysis (PCA) and cluster heat map analysis, with the selection of the first two principal components for plotting. PC1 and PC2 accounted for 46.6% and 30.8% of the variance, respectively, indicating excellent performance of the new model. The results demonstrated a significant separation between the control and treatment groups, as both the CK and T150 groups were located on the upper side of the *X*-axis (Figure 1A). Moreover, cluster heat map analysis revealed that the CK and T150 groups clustered together (Figure 1B).

### 3.2. Metabolite Type and Quantity Analysis

After widely targeted metabolomics analysis, a total of 862 metabolites belonging to 18 different categories were detected from cotton plants subjected to three concentrations of NaCl stress. Among them, the following 11 categories accounted for more than 80% of the identified metabolites: terpenoids (119 types; 13.8%), amino acids (99 types; 11.5%), flavonoids (81 types; 9.4%), sugars and alcohols (71 types; 8.2%), lipids (70 types; 8.1%), organic acids (65 types; 7.5%), alkaloids (61 types; 7.1%), polyphenols (45 types; 5.2%), nucleotides (38 types; 4.4%), ketones, aldehydes and acids (34 types; 3.9%), and steroids (25 types; 2.9%) (Figure 1C).

### 3.3. Analysis of Cotton Metabolites

**Figure 2:**
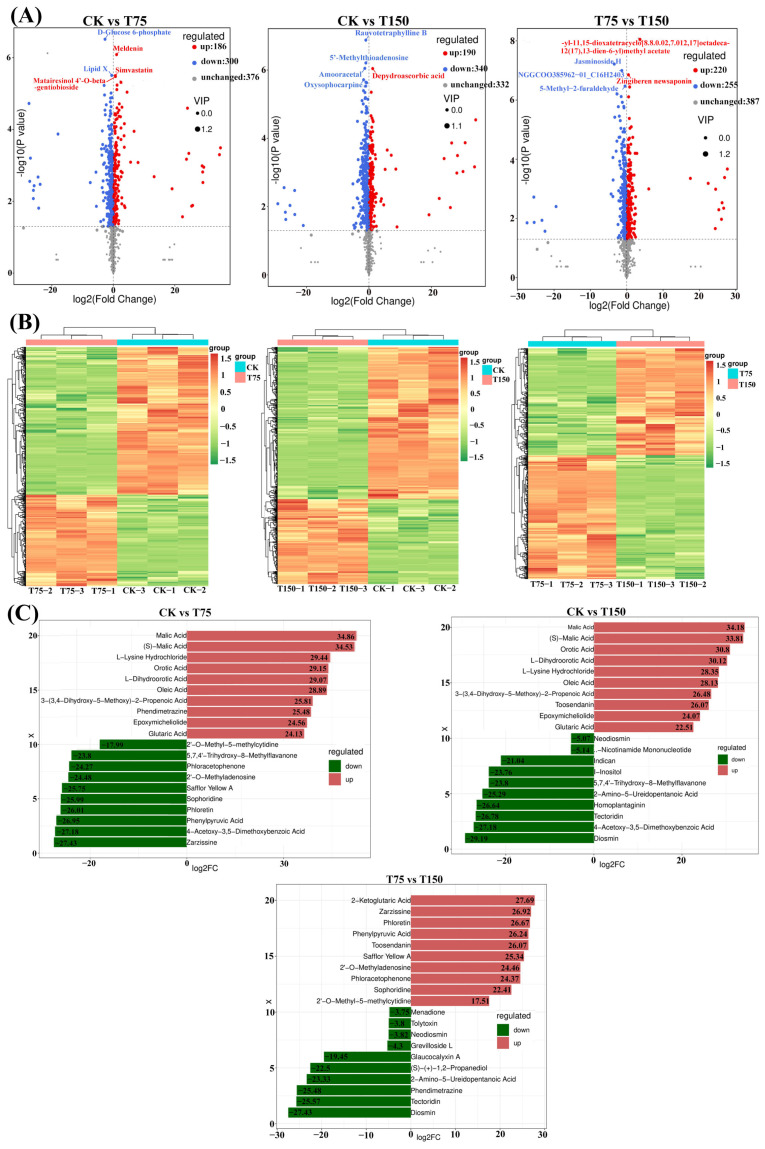
Up- and downregulation of DMs in different treatment groups. (**A**) Volcano plots showing upregulated and downregulated metabolites. (**B**) Heatmap showing up- and downregulated metabolites. (**C**) Up- and downregulation of the top ten DMs.

Through analyzing differential metabolites (DMs) and drawing the volcano plots (Figure 2A), there were significant differences in 486 metabolites between the CK group and the T75 group. Among these, 186 metabolites showed upregulation, while 300 metabolites exhibited downregulation. Furthermore, 530 differential metabolites were identified between the CK and T150 groups, including 190 upregulated and 340 downregulated metabolites. Furthermore, there were 475 differential metabolites between the T75 and T150 groups, with 220 upregulated and 255 downregulated metabolites observed. The changes in these differential metabolites were clearly demonstrated by the cluster heatmap (Figure 2B). Finally, we presented the top 10 substances that were significantly up- or downregulated based on fold change values (Figure 2C).

### 3.4. KEGG Enrichment Analysis of Differential Metabolites

**Figure 3:**
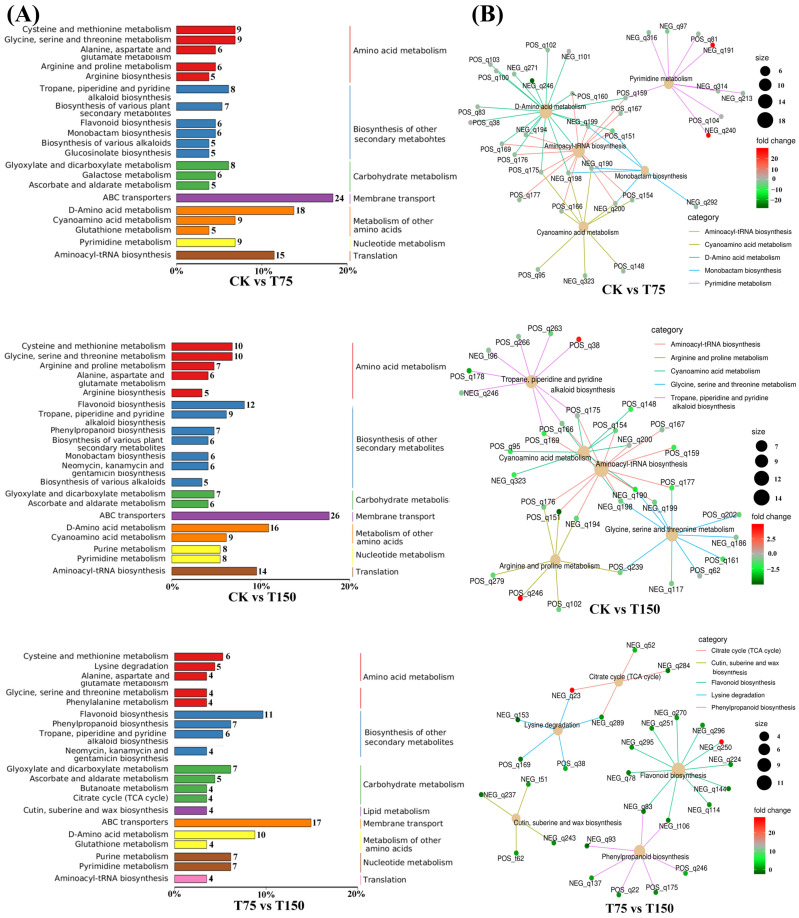
Differential metabolite enrichment pathways and their correlation analysis. (**A**) Differential metabolites were enriched KEGG pathway in the CK vs. T75, CK vs. T150, T75 vs. T150 groups. (**B**) The correlation analyzed the top five metabolic pathways and differential metabolites in the CK vs. T75, CK vs. T150, T75 vs. T150 groups.

We classified the differential metabolites based on KEGG annotation using the KEGG database and found that these metabolites were mainly enriched in amino acid metabolism, biosynthesis of secondary metabolite, and carbohydrate metabolism (Figure 3A). Subsequently, we performed KEGG enrichment analysis on these differential metabolites and found their significant enrichment in primary metabolic pathways such as amino acids during the initial stage of salt stress. With the salt concentration increased, there was a shift in the differential metabolites from amino acid metabolism to secondary metabolism pathways like flavonoid biosynthesis (Figure 3B).

### 3.5. K-Means Cluster Analysis of Differential Metabolites

**Figure 4:**
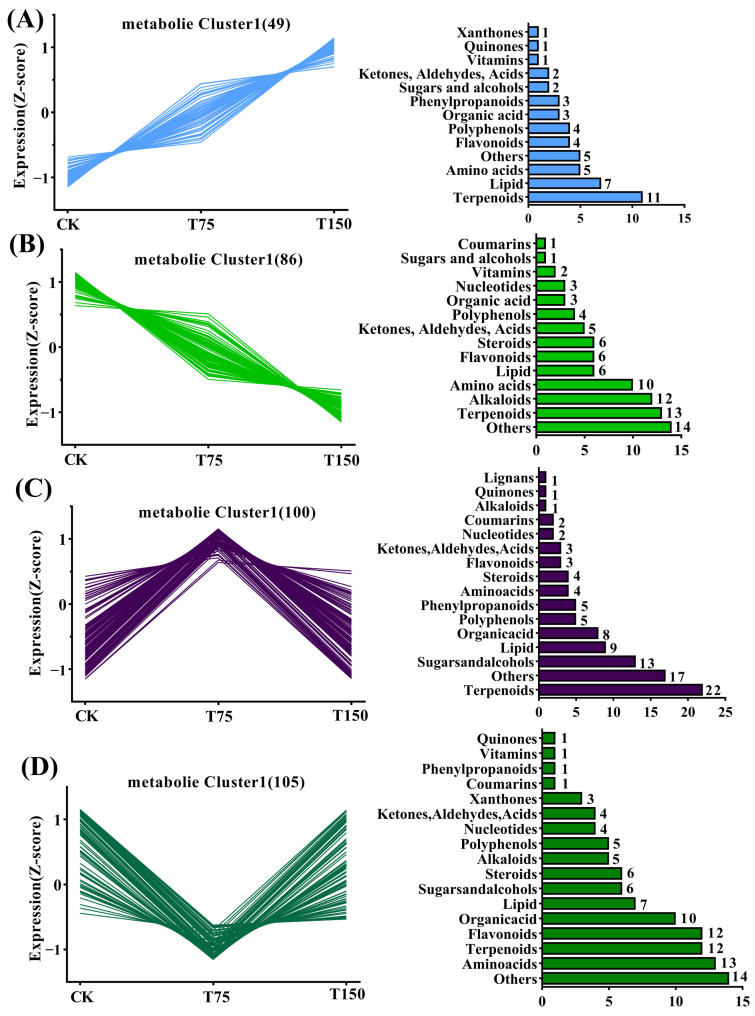
K-means clustering analysis of 0, 75, and 150 mM NaCl differential metabolites showed four changes. (**A**) Continuous upregulation. (**B**) Continuous downregulation. (**C**) first upregulation and then downregulation. (**D**) first downregulation and then upregulation.

To investigate the response of different metabolites to NaCl stress at concentrations of 0, 75, and 150 mM, K-means clustering analysis was performed on 340 coexpressed differential metabolites (Figure 4). The results showed that these metabolites could be grouped into four classes comprising 49, 86, 100 and 105 members, respectively. Among them, class 1 exhibited a consistent upregulation in substance content with the increase in NaCl concentration (Figure 4A), while class 2 showed a consistent downregulation trend. Classifying the main metabolites within each group revealed that terpenoids and lipids were predominant in class 1, whereas terpenoids and alkaloids were predominant in class 2 (Figure 4B). In contrast, class 3 displayed an initial up- and downregulated pattern with significant representation of terpenoids, sugars, and alcohols as major constituents (Figure 4C); similarly for class 4, it demonstrated an initial downregulation followed by the upregulated trend primarily driven by amino acids and terpenoids (Figure 4D).

### 3.6. Effects of Salt-Stressed Cotton Plants on A. gossypii

**Figure 5:**
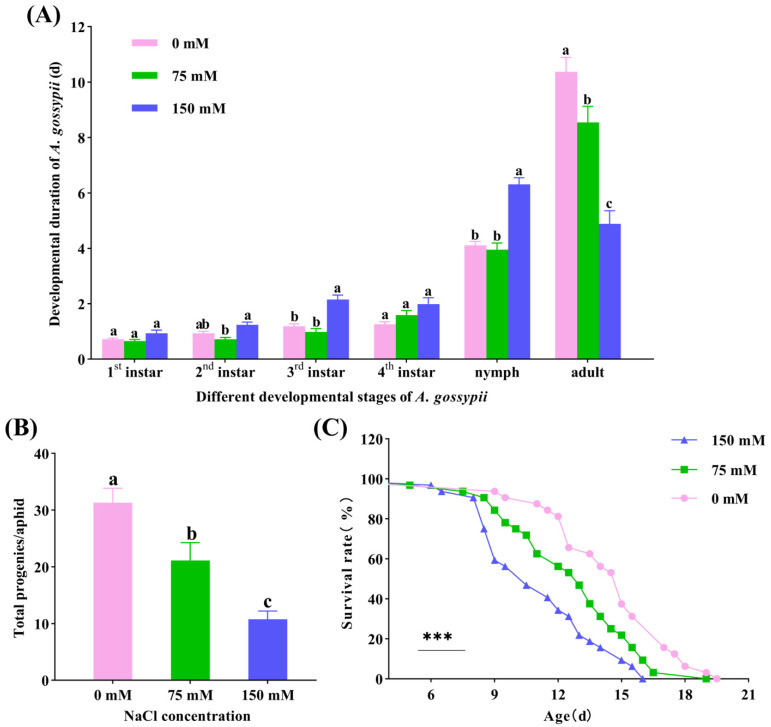
Effects of cotton plants under NaCl stress with different concentrations on nymphal duration, adult longevity (**A**), fecundity (**B**) and survival rate (**C**) of *Aphis gossypii* Glover. Survival statistics were calculated using the Kaplan–Meier survival curve and compared using the log-rank test (individuals = 30, *** *p* < 0.001). Different letters indicate significant differences among treatments (*p* < 0.05).

We investigated the effects of cotton plants under 0, 75, and 150 mM NaCl stress on various parameters including nymphal duration, adult longevity, fecundity, and survival rate of cotton aphids. The duration of nymphal aphids significantly extended to 6.31 days under 150 mM NaCl treatment when compared to the control group (0 mM NaCl, 4.10 days) (*F* = 20.55, *df* = 2, *p* < 0.001). In addition, compared with the 75 mM NaCl treatment group (0.71 days), the second instar nymph duration in the 150 mM NaCl treatment group significantly extended to 1.23 days (*X*^2^ = 16.32, *df* = 2, *p* < 0.001). Similarly, the third instar nymph duration significantly prolonged from 1.19 days (0 mM NaCl) to 2.16 days (150 mM NaCl) (*X*^2^ = 33.93, *df* = 2, *p* < 0.001). Meanwhile, the longevity of adult aphids decreased significantly under 75 and 150 mM NaCl treatments, with an average of 10.38 days (0 mM NaCl) reduced to 8.55 and 4.89 days, respectively (*F =* 28.30, *df* = 2, *p* < 0.001) (Figure 5A). Moreover, the total reproduction number of single females decreased significantly from 31.31 (0 mM NaCl) to 21.13 (75 mM NaCl) and 10.75 (150 mM NaCl) (*F* = 18.63, *df* = 2, *p* < 0.001) (Figure 5B). The shortest survival time of *A. gossypii* in 150 mM NaCl treatment group was 16 days, while the longest survival time of *A. gossypii* in 0 mM NaCl treatment group was 19.5 days. The survival rate of aphids decreased from 81.25% (0 mM NaCl) to 56.25% (75 mM NaCl) and 34.38% (150 mM NaCl) on the 12th day. In addition, at 16 days, the survival rate of *A. gossypii* in the 150 mM NaCl group was 0%, while those in the 0 and 75 mM NaCl groups were 31.25% and 9.375%, respectively (*X*^2^ = 20.66, *df* = 2, *p* < 0.001) (Figure 5C).

### 3.7. The Correlation Analysis between Fitness and Differential Metabolites

By analyzing the correlation between the fitness-related parameters of cotton aphids and the differential metabolites in cotton plants under 0, 75, and 150 mM NaCl concentration, 49 continuously upregulated substances were significantly negatively correlated with the longevity and fecundity of cotton aphids, while 86 continuously downregulated substances exhibited the opposite trend (Table 1).

## 4. Discussions

The impact of environmental stress on phytophagous pests is believed to be mediated through the modulation of plant metabolism [27,28]. In this study, we observed alterations in amino acid metabolism, secondary metabolite biosynthesis, and carbohydrate metabolism in cotton plants under salt stress conditions. Furthermore, mild salt stress primarily affected primary metabolic pathways such as amino acid metabolism, while moderate salt stress predominantly impacted secondary metabolic processes like flavonoid metabolism. These results are in agreement with those reported by Han et al. [20]. Moreover, we observed that moderate salt stress significantly prolonged the developmental duration of nymphal aphids and significantly reduced the longevity and fecundity of adult aphids under mild and moderate salt stress, which is consistent with the results of Wang et al. [25]. This shows that, in salinized soil, the effective cotton planting cycle can reduce the reproductive generation and population density of cotton aphids, which has great practical significance for reducing the harm of cotton aphids on cotton plants and improving cotton quality.

Amino acids and their derivatives play important roles in plant resistance. This study revealed a notable increase in amino acid levels, aligning with the findings of Guo et al. [29]. Proline plays a vital role in enhancing plant resilience to salt stress by regulating cell osmotic capacity, maintaining cytoplasmic pH balance, stabilizing proteins, and neutralizing reactive oxygen radicals [30,31]. Notably, this research observed a notable increase in the proline levels of cotton plants under moderate salt stress, which is consistent with the studies conducted by Han et al. [20] and Guo et al. [32]. The accumulation of other amino acids may be attributed to protein degradation and the inhibition of protein synthesis [33,34]. Furthermore, aspartate, glutamate, and glutamine serve as crucial nitrogen storage and transport compounds in plants, with their accumulation closely associated with nitrogen availability [35,36,37]. Meanwhile, nitrogen represents a primary limiting resource for phytophagous pests, whereby reduced plant nitrogen nutrition affects the population abundance of these pests as well as the extent of damage inflicted upon the host plants [38,39]. Notably, this study observed a decreased level of glutamate and glutamine in cotton plants under salt stress conditions, which may indicate a reduced ability to assimilate nitrogen. Therefore, the decrease in nitrogen content within salt-stressed cotton plants may be one of the important factors affecting the growth, development, and fecundity of cotton aphids.

Secondary metabolites, such as polyphenols, flavonoids, and tannins, play a crucial role in plant adaptation to salt stress and defense against phytophagous pests. These metabolites exert their effects by interacting with free radicals, chelating metal ions, and scavenging oxygen [40,41,42]. Certain aromatic amino acids including phenylalanine, tryptophan, and tyrosine serve as precursors for the synthesis of natural substances such as alkaloids, flavonoids, auxin, and cell wall elements that are crucial for plant development and response to environmental stresses. In this study, it was observed that the expression of these amino acids decreased, whereas secondary metabolites like alkaloids and flavonoids increased [43,44], which is consistent with the results of Han et al. [20] and Ma et al. [23]. Furthermore, secondary substances like flavonoids, alkaloids, and polyphenols, play a role in plant defense against pests, markedly influencing the growth and development of pests [45]. Under salt stress, cotton plants exhibited increased levels of quercetin, potentially hindering the growth of *Spodoptera litura* and *Helicoverpa armigera* larvae, leading to weight reduction [46,47]. The presence of gossypol and tannins in cotton leaves adversely impacts herbivorous pests [48,49]. Plant-derived tannins, known for their bitter taste, exhibit high toxicity against phytophagous pests and shield plants from them [50,51,52]. Incorporating tannins into feed demonstrated potent food-repelling properties and hindered the growth of cotton bollworms [53]. Gossypol significantly inhibits the growth of cotton aphids, reduces the net weight of cotton bollworms, and affects the plumage rate of adults [54,55,56]. Elevated levels of gossypol impacted the breeding and growth of *Bemisia tabaci* by reducing nymphal survival rates, population growth, nymphal development periods, and adult fertility [57,58]. Consequently, the increase in certain secondary metabolites may be associated with the reduced fitness of cotton aphids in cotton plants under salt stress conditions.

Carbohydrates play a pivotal role as the primary energy source in plant metabolic processes and serve as essential structural components of cells, crucial for sustaining osmotic equilibrium, neutralizing reactive oxygen species (ROS), regulating intracellular homeostasis, and modulating stress response mechanisms [59,60]. Glucose supplementation enhances peroxidase and superoxide dismutase activities in plants, facilitating the elimination of excessive levels of malondialdehyde, superoxide radicals, and hydrogen peroxide induced by salt stress conditions. Additionally, the presence of glucose can elevate chlorophyll levels in plants, thereby improving photosynthetic efficiency [61]. The introduction of glucose has been found to promote antioxidant enzyme activity and regulate nitrogen metabolism to safeguard chloroplast ultrastructure, thereby improving a cucumber’s tolerance to salt stress [62,63]. This research revealed an increase in glucose, sucrose, xylose, and β-D-fructose 2-phosphate levels in cotton plants under salt stress, which is consistent with the results of Han et al. [20]. Nonetheless, saccharides, the primary nutrients for cotton aphids, showed a notable inverse correlation with their growth, development, and reproductive capacity. The enhanced resistance of cotton plants under salt stress conditions against cotton aphids may be attributed to elevated saccharide concentrations within their tissues. This could potentially impact the growth and maturation of cotton aphids by amplifying their metabolic pressure for saccharide release [64,65].

In conclusion, our study provides comprehensive insights into the impact of continuous salt stress on the physiological metabolism of cotton plants and its consequential effects on the growth, development, and fecundity of cotton aphids. These results demonstrate that salt stress induces significant alterations in the metabolic pathways of cotton plants. Therefore, we argued that moderately saline soil can partially inhibit the development of cotton aphids on cotton plants, thereby mitigating their detrimental effects. In addition, we conducted a thorough analysis on the correlation between the different metabolites in cotton plants and the fitness data pertaining to cotton aphids under salt stress conditions. A total of 49 substances were found to exhibit significant correlations with the resistance of cotton plants against cotton aphids. These substances include flavonoids such as dihydromyricetin, neoastilbin, and taxifolin; polyphenols such as gossypol, olivetol, and glucosyringic acid; and terpenoids such as chloranthelactone E, inugenol, and polpunonic acid. It is plausible that these substances may contribute to the negative effect of salt-stressed cotton plants on the fitness of cotton aphids. However, it is urgent to investigate how these substances affect the fitness of cotton aphids, as well as the underlying molecular mechanisms by which cotton plants under salt stress conditions influence their growth, development, and fecundity.

## 5. Conclusions

In summary, cotton metabolism had significant differences under three different NaCl levels. Moreover, as the concentration of NaCl increased, there was a significant decline in the fecundity, adult longevity, and survival rate of cotton aphids in NaCl-stressed cotton plants. These results support the hypothesis that plants growing under salt stress are better defended against herbivores. In addition, we observed a substantial upregulation of 49 metabolites and downregulation of 86 metabolites in cotton plants with increasing NaCl concentration, which exhibited a strong correlation with the growth, development, and fecundity of cotton aphids. Therefore, these substances in cotton leaves may serve as crucial mediators influencing the growth and development of cotton aphids.

## Figures and Tables

**Table 1:** Correlation coefficient between the first 10 continuously up- and downregulated differential metabolites and the adult longevity and fecundity on cotton plants under 0, 75, and 150 mM NaCl stress.

ID	Differential Metabolites	Correlation Coefficient R	
		Adult Longevity	Fecundity/Aphid
NEG_q114	Dihydromyricetin	−0.969	−0.915
NEG_q228	Neoastilbin	−0.944	−0.985
NEG_q295	Taxifolin	−0.914	−0.975
NEG_q156	Gossypol	−0.903	−0.946
NEG_q238	Olivetol	−0.882	−0.956
NEG_q152	Glucosyringic Acid	−0.948	−0.893
NEG_q82	Chloranthalactone E	−0.852	−0.967
NEG_q164	Incensole	−0.903	−0.977
NEG_q262	Polpunonic Acid	−0.879	−0.965
NEG_t44	Centellasaponin B	−0.963	−0.95
NEG_t136	Benzyl glucoside	0.984	0.94
NEG_t42	Microcystin-LR	0.966	0.981
NEG_t122	Rauvotetraphylline B	0.965	0.972
NEG_t125	Eupteleasaponin I	0.963	0.981
POS_t112	Cis-Jasmone	0.952	0.985
NEG_q78	Catechin	0.951	0.927
POS_q127	Histamine (Phosphate)	0.951	0.976
POS_q237	Sah	0.949	0.915
NEG_q138	Fraxin	0.948	0.982
POS_q279	γ-Aminobutyric acid	0.944	0.968

## Data Availability

The data presented in this study are available on request from the corresponding author.
